# How to describe species richness patterns for bryophyte conservation?

**DOI:** 10.1002/ece3.1796

**Published:** 2015-10-28

**Authors:** Helena Hespanhol, Katia Cezón, Ángel M. Felicísimo, Jesús Muñoz, Rubén G. Mateo

**Affiliations:** ^1^ CIBIO/InBioCentro de Investigação em Biodiversidade e Recursos Genéticos da Universidade do PortoCampus Agrário de Vairão4485‐661VairãoPortugal; ^2^ Real Jardín Botánico (CSIC)Plaza de Murillo 228014MadridSpain; ^3^ Centro Universitario de MéridaUniversidad de Extremadura06800MéridaSpain; ^4^ Department of Ecology & EvolutionUniversity of LausanneBiophore Building1015LausanneSwitzerland; ^5^ Universidad Tecnológica IndoaméricaBolívar 20‐35AmbatoEcuador

**Keywords:** Biodiversity conservation, biological collections, bryophytes, richness models, species distribution models

## Abstract

A large amount of data for inconspicuous taxa is stored in natural history collections; however, this information is often neglected for biodiversity patterns studies. Here, we evaluate the performance of direct interpolation of museum collections data, equivalent to the traditional approach used in bryophyte conservation planning, and stacked species distribution models (S‐SDMs) to produce reliable reconstructions of species richness patterns, given that differences between these methods have been insufficiently evaluated for inconspicuous taxa. Our objective was to contrast if species distribution models produce better inferences of diversity richness than simply selecting areas with the higher species numbers. As model species, we selected Iberian species of the genus *Grimmia* (Bryophyta), and we used four well‐collected areas to compare and validate the following models: 1) four Maxent richness models, each generated without the data from one of the four areas, and a reference model created using all of the data and 2) four richness models obtained through direct spatial interpolation, each generated without the data from one area, and a reference model created with all of the data. The correlations between the partial and reference Maxent models were higher in all cases (0.45 to 0.99), whereas the correlations between the spatial interpolation models were negative and weak (−0.3 to −0.06). Our results demonstrate for the first time that S‐SDMs offer a useful tool for identifying detailed richness patterns for inconspicuous taxa such as bryophytes and improving incomplete distributions by assessing the potential richness of under‐surveyed areas, filling major gaps in the available data. In addition, the proposed strategy would enhance the value of the vast number of specimens housed in biological collections.

## Introduction

Cryptogams, invertebrates, and other inconspicuous groups are of great importance in the functioning of ecosystems (Hafernik 1992; Vanderpoorten and Goffinet 2009). But, they have not received as much attention as charismatic taxonomic groups, such as flowering plants, mammals, or birds, for the establishment of conservation measures or the study of biodiversity patterns (Oliver and Beattie 1993; Hunter and Webb 2002). Due to the increasing pressure on the natural environment, many individual species and sites that are important for biodiversity conservation now face increasing threats (Brooks et al. 2002; Butchart et al. 2010). The estimation of species richness distribution is necessary to understand spatial patterns of biodiversity (Ricklefs 2004), to establish conservation strategies (Bombi et al. 2011), or to predict future patterns of biodiversity under global change (Algar et al. 2009).

However, information on species richness is often incomplete, especially for inconspicuous organisms due to several reasons, including insufficient field surveys for most geographic areas, strong spatial biases in the survey data (Sérgio and Draper 2002; Wilson et al. 2005), or a lack of taxonomic knowledge about such “forgotten” groups (the “taxonomic impediment”) (Carvalho et al. 2007). Also, this is in part because the inclusion of invertebrates and nonflowering plants is perceived as being too time‐consuming, costly, and difficult because of the shortage of specialists (Oliver and Beattie 1993). Finally, records of vertebrates or vascular plants are recurrently used as surrogates for estimates of total biodiversity. But, for example, bryophytes are characterized by high dispersal capacities (Muñoz et al. 2004) and strikingly different ecophysiological strategies as compared to flowering plants (Vanderpoorten and Goffinet 2009). As a result, the existence of a spatial congruence in species richness distribution patterns between flowering plants and bryophytes has been questioned (Shaw et al. 2005; Hedenäs 2007; Geffert et al. 2013). So, the use of charismatic taxonomic groups as indicators of total biodiversity should be revised and new techniques should be applied to know the richness patterns of inconspicuous groups. To overcome the lack of planned data sampling, natural history collections (NHCs) represent a useful solution. These sources of data have some advantages, including quantity, accessibility, taxonomic confidence, frequent updating, and wide temporal and geographic scales (Graham et al. 2004; Garcillán and Ezcurra 2011) and therefore are useful for conservation purposes (Loiselle et al. 2003; Gaubert et al. 2006; Sérgio et al. 2007b; Newbold 2010).

The simplest modeling process that can be applied to a collection of observations is based on interpolation of known locations and expert knowledge (Boitani et al. 2011). A number of studies have directly used raw species distribution data or distribution maps derived from simple forms of spatial interpolation that estimate unknown data from neighbor values (i.e., direct interpolation of data) to evaluate patterns of biodiversity for conservation assessment (Ferrier 2002; Graham and Hijmans 2006; Hernandez‐Stefanoni and Ponce‐Hernandez 2006; Sérgio et al. 2012; Geffert et al. 2013). Historically, atlas works, which aim at mapping species distributions at large spatial scales, have been based on the collection of observations and their representation on a continuous spatial grid through interpolation techniques (Franklin and Miller 2009). This intuitive and simple approach that consists on aggregating the NHCs data and transferring the resulting information to a geographic space may be useful for coarse‐scaled conservation assessments, and it appears as reasonable if data are spatially well distributed and the density of locations high (Ferrier 2002).

As the recording effort cannot be increased very much in most cases (Vanderpoorten et al. 2005), an alternative is to use the available NHCs to generate species distribution models (SDMs). Such tools integrate the relationships between data on species distributions available in NHCs and meaningful environmental variables to build the habitat suitability of the species (Mateo et al. 2011). The approach of stacking individual species distribution models (S‐SDMs) to generate maps of potential richness (Guisan and Rahbek 2011) has become widely used in conservation planning and the design of reserve networks, for example, if the final aim is preserving the most unique and biodiverse areas (Margules and Pressey 2000; Myers et al. 2000; Mateo et al. 2013a), or the identification of suitable areas for threatened or otherwise rare species (Austin et al. 1996; Thomas et al. 2004; Graham and Hijmans 2006; Jeschke and Strayer 2008), and could be a useful tool to integrate poorly known and inconspicuous groups into the process of designing priority areas. The S‐SDM approach considers a simple stacking of individual species responses to the environment and therefore does not explicitly integrate any potential constraint on the maximum number of species that can co‐occur in a given area (e.g., available energy, heterogeneity within the modeled unit, or biotic interactions) (Guisan and Rahbek 2011). Few examples of SDMs applied to bryophytes can be found in the bibliography (Kruijer et al. 2010; Sérgio et al. 2011; Désamoré et al. 2012; Roux et al. 2012; Mateo et al. 2013b) in comparison with other organisms (e.g., vascular plants, birds, and mammals), and none of these studies has been applied yet to investigate species richness patterns.

The conservation of bryophytes is behind that of flowering plants (Schumacker and Martiny 1995; Vanderpoorten et al. 2005), although they are subject to many of the threats that flowering plants face. There are a number of programs which aim is to identify and protect a network of the best sites for biodiversity conservation (e.g., http://www.cbd.int; http://www.natura.org), based on biodiversity richness. One of them is the Important Plant Areas (IPA) program (http://www.plantlife.org.uk), focused on the identification of priority areas for wild plants, fungi, and their habitats around the world and to ensure their long‐term survival. It offers guidelines to identify and protect regions with high diversity in habitats and species based on consistent criteria (Anderson 2002). Connected with the IPA project, some others programs have been developed, as the Important Bryophyte Areas or “IbrA” (Papp 2008). These programs offer the possibility to protect and properly manage the priority conservation sites, but one of the basic requirements is that the design of such networks must be based on sound knowledge on species' distributions and typically uses species distribution maps based on raw occurrence data. Overall, the traditional approach on bryophyte conservation planning is to collect all the available data, generally based on NHC, and after an implicit spatial interpolation process, to propose the richest areas as priority areas for conservation (Infante and Heras 2012; Sérgio et al. 2012).

In this article, we compare the traditional method used in bryophyte conservation planning to estimate richness patterns that aggregates raw distribution data and transfers the resulting information to a geographic space, and the widely method of generating SDMs that is being used in conservation planning and the design of reserve networks, by integrating the relationships between data on species distributions available in NHCs and meaningful environmental variables to build the habitat suitability of the species. We evaluate these two alternative approaches with different complexity to estimate species richness patterns (direct interpolation of data vs. S‐SDMs) using museum collections data, given that differences between these methods have been insufficiently evaluated for inconspicuous taxa. Here, we use a spatial interpolation model as an equivalent approach to the traditional method commonly used in bryophyte conservation planning which consists on aggregating the NHC data and transferring the resulting information to a geographic space. Specifically, we investigate how these approaches can influence richness patterns, how much are affected by the data, and how this variation influences inferences drawn from these richness maps. Here, we also assess whether accurate predictions of potential richness can be achieved for bryophytes using S‐SDMs, as little is known about the performance of S‐SDMs in inconspicuous groups. In bryophytes, climatic filters and long‐distance dispersal have indeed been traditionally assumed to shape species composition at the continental scale (Muñoz et al. 2004), so that macroclimatic factors proved excellent predictors of bryophyte‐dominated ecosystems and bryophyte species distributions. Therefore, they are a good subject of study for SDMs (Sérgio et al. 2011; Mateo et al. 2013b). To perform this study, we used the genus *Grimmia* as models species because the taxonomy of this genus is well known, and thus, we can avoid the “taxonomic impediment” mentioned above. Moreover, the group has been relatively well collected in the Iberian Peninsula. This combination of factors makes the group ideal for testing our hypothesis: When species richness patterns are derived from direct interpolation of distributional data on inconspicuous taxa housed in NHCs, these data by themselves are not sufficiently informative to derive richness patterns. To test our hypothesis, we sought answer to several questions. Do S‐SDMs generated from NHCs produce the same outcome, in terms of richness, as direct interpolation models of the information contained in the NHCs for inconspicuous taxa, namely bryophytes? Do S‐SDMs generated from NHCs produce reliable results for inconspicuous taxa?

## Materials and Methods

### Study area and species data

The genus *Grimmia* is an important component of the Iberian Peninsula bryoflora with regard to both the number of species and their distribution and ecological significance. The group was revised for several geographic areas by one of the authors (JM) who has studied most of the Iberian specimens deposited in herbaria worldwide. The genus includes 31 species from the Iberian Peninsula (Muñoz and Pando 2000; Casas et al. 2006; Muñoz et al. 2009). Thirteen species of the genus are known from fewer than five Iberian localities and have been excluded from this study (*G. anomala*,* G. arenaria*,* G. atrata*,* G. capillata*,* G. crinitoleucophaea*,* G. elatior*,* G. elongata*,* G. horrida*,* G. incurva*,* G. longirostris*,* G. mollis*,* G. muehlenbeckii,* and *G. unicolor*). The taxonomic status of *G. dissimulata* and *G. meridionalis* is currently under study using molecular methods, and these two species are not discussed further in this study.

Information is available about the conservation status of the species included in this study at different scales and in different areas. *G. caespiticia* (Brid.) Jur. and *G. atrata* Hornsch. are considered rare in Europe (Schumacker and Martiny 1995). At the scale of the Iberian Peninsula, *G. arenaria* Hampe, *G. crinitoleucophaea* Cardot*, G. mollis* Bruch & Schimp., *G. muehlenbeckii* Schimp., and *G. unicolor* Hook. are considered vulnerable. *Grimmia capillata* De Not. is data deficient‐new, *G. incurva* Schwägr. is near threatened, and the remaining species are included in the list of species of least concern and attention (Sérgio et al. 2007a).

**Figure 1:**
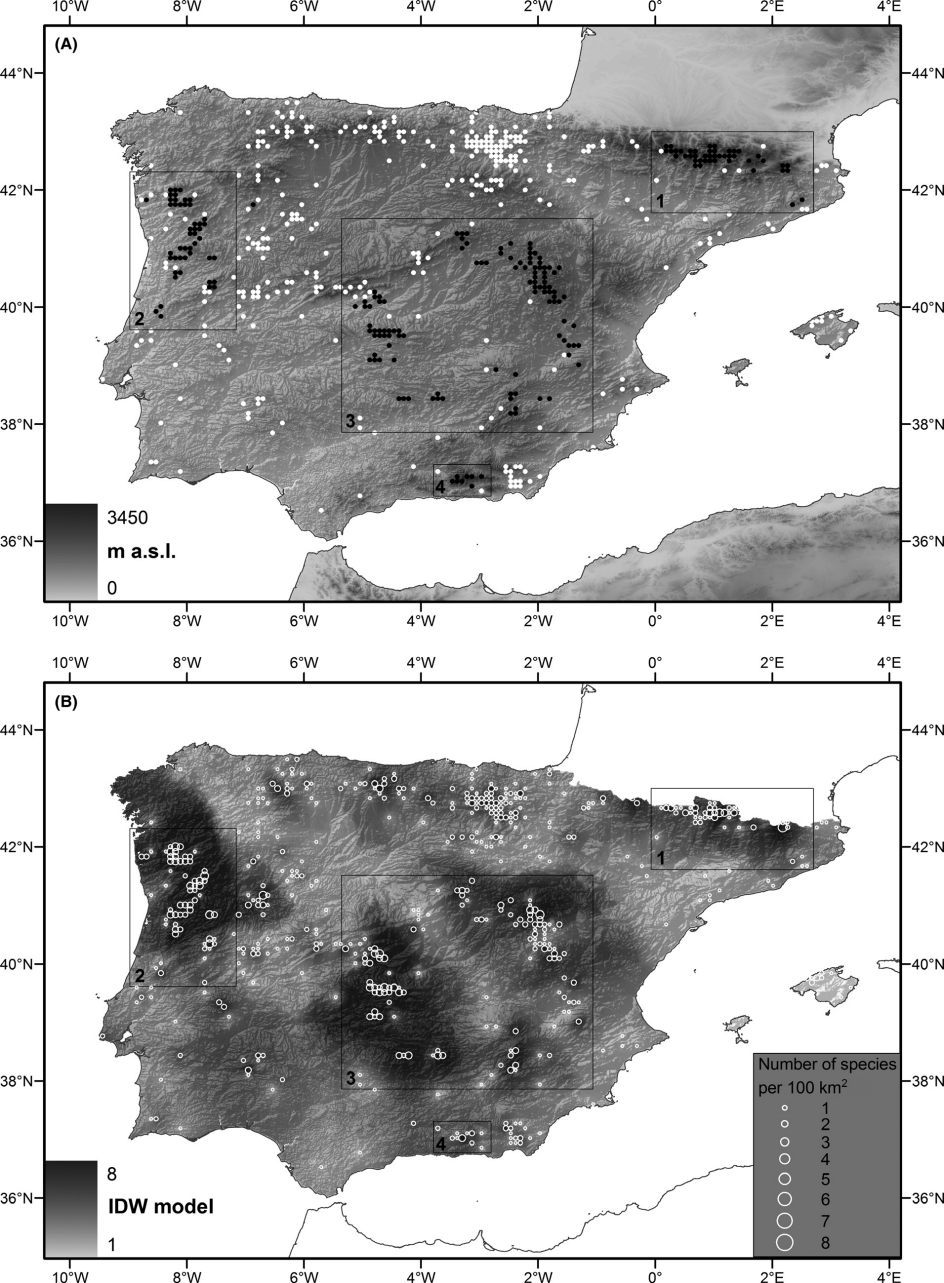
Collection localities resampled at 10 × 10 km pixel size. 1 – Creu Casas' extensive and intensive collections across the Pyrenees and Francisco Lloret's PhD; 2 – PhD thesis data of Helena Hespanhol (NW Portugal); 3 – PhD thesis data of Katia Cezón (Castilla‐La Mancha); 4 – PhD thesis data of Susana Rams (Sierra Nevada). (A) The background represents the digital elevation model, white dots opportunistic sampling localities, and black dots systematically sampled areas. (B) The background represents the number of *Grimmia* species according to an Inverse Distance Weighting (IDW) interpolation using information from all of the collection localities. The number of different *Grimmia* species per collection locality is represented by graduated circles.

We mapped the collections on a 0.01° (~0.72 km^2^ in the study area) grid, the spatial resolution used for the study. At this spatial resolution, certain specimens collected in close proximity coincide in the same pixel and represent a single presence. The collection localities were resampled at a pixel size of 10 × 10 km to avoid the overrepresentation of very low numbers in the interpolation analyses (Fig. 1A). We employed two different data sets, the white dots in Figure 1A represent opportunistic sampling localities, whereas the black dots indicate systematically sampled localities, corresponding to the following data sets: (1) Creu Casas' extensive and intensive collections across the Pyrenees and Francisco Lloret's PhD dissertation; (2) the PhD thesis data of Helena Hespanhol (NW Portugal); (3) the PhD thesis data of Katia Cezón (Castilla‐La Mancha); and (4) the PhD thesis data of Susana Rams (Sierra Nevada).

### Interpolation model

**Figure 2:**
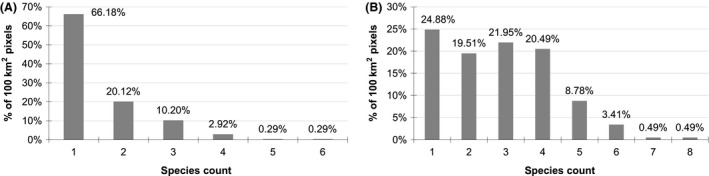
Percentage of collection localities (100 km^2^ pixels) by *Grimmia* species count for (A) opportunistic sampling localities and (B) systematically sampled localities.

We generated direct interpolation models to predict potential richness and to be compared with S‐SDMs, using the inverse distance weighting (IDW) technique in ArcGIS 9.3 (ESRI, Redlands, California, USA). Only one or two species had been collected from most of the collection localities, irrespective of the number of specimens, which may reflect the fact that most of the collections were opportunistic and only detected the most common and conspicuous species (Fig. 2). For this reason and to avoid the overrepresentation of very low numbers in the interpolation analyses, we defined a fishnet of a 10 × 10 km cell size over the Iberian Peninsula and counted the number of different species occurring in each cell. This aggregated data set was interpolated using IDW with a radius of 12 and a power of 1.6 to reduce the influence of the closest points, thus producing a smoother final surface (Fig. 1B).

### Stacked species distribution models (S‐SDMs)

To generate the different SDMs described below, we used Maxent, one of the modeling technique most used in the literature, as it provides consistently reliable results in the work performed on the comparison of other modeling techniques (Elith et al. 2006). It is a machine‐learning technique based on the principle of maximum entropy (Phillips et al. 2006; Phillips and Dudík 2008; Elith et al. 2011). This technique seeks a marginal suitability function for each variable that matches the empirical data, is maximally uninformative (close to the uniform distribution) elsewhere, and has a mean equal to that of the empirical data (Warren and Seifert 2011). Because the requirement of equal means can produce the undesirable result of overfitting the data used to train the model, Maxent has a regularization multiplier (β) that can tune the model to avoid such overfitting. In this study, we used Maxent 3.3.3e (MAXENT; Phillips et al. 2006, software available at https://www.cs.princeton.edu/~schapire/maxent/) with only the convergence threshold (0.00001) and the number of background points (10,000) set to their default values. To avoid overfitting, we increased the regularization multiplier (β = 2). This choice produced less open‐ended response curves.

The nineteen Worldclim 1.4 bioclimatic variables at spatial resolution of about 1 × 1 km^2^ (http://www.worldclim.org) were used as independent variables. These bioclimatic variables result from the global land area interpolation of the climate point data for the period 1950–2000 (Hijmans et al. 2005). They were intersected with the presence data and with 10,000 points selected at random and used as the background data set. To avoid multicollinearity, we performed a correlation analysis on the background data set and eliminated one of the variables in each pair that showed a Pearson correlation value >0.8. The final data sets included Isothermality (bio_03), Temperature Annual Range (bio_07), Mean Temperature of Warmest Quarter (bio_10), Mean Temperature of Coldest Quarter (bio_11), Precipitation Seasonality (bio_15, coefficient of variation), Precipitation of Wettest Quarter (bio_16), and Precipitation of Driest Quarter (bio_17). Because *Grimmia* species are sensitive to the chemical characteristics of the rocks on which they grow, we also included a soil acidity/alkalinity categorical variable that was derived from the European Soil Database v2.0 (Data S1).

The performance of the Maxent models was evaluated using the area under the curve (AUC) of the receiver operating characteristic (ROC) curve. Although the validity of this statistic as a technique for evaluating models has recently been challenged (Lobo et al. 2008; Peterson et al. 2008), particularly for presence‐only data, its use in contexts similar to the present study has also been justified (Phillips et al. 2006; Anderson and Gonzalez 2011). For each species, the AUC was calculated through cross‐validation based on 10 replicates, and the final model was the average of the replicates.

There are many ways to generate richness maps from original models (Ferrier and Guisan 2006). Following Calabrese et al. (2014), we summed the original values obtained for each individual taxon without reclassification for presence/absence. The resulting richness model can be considered as an estimate of the potential number of species that could be present in each particular pixel (Gelfand et al. 2005; Wilson et al. 2005).

### Validation and comparison of the richness models

We used IDW to create four partial richness models for the entire Iberian Peninsula. Each partial model excluded one of the four systematically sampled data sets (Fig. 1B). We also used IDW to create a reference model using all of the available presence data. In addition, we generated four partial S‐SDMs, each of which excluded the data for one of those four systematically sampled data sets, and we generated a reference model using all of the available presence data. For the partial models, the number of unique presences is indicated in Table 1; duplicate presences were removed in all cases. Partial models were generated with the aim to test the predictive power of the models (IDW and S‐SDMs) in each of the four systematically sampled areas. Following Hernandez et al. (2006), the four partial models were compared with the reference model, considered to be the most representative of the true distribution of the species given the limitations of the modeling method, the species occurrence, and environmental data available. Lastly, for each of the four systematically sampled areas (i.e., setting the analysis window to only that area) and separately for IDW and S‐SDMs, we calculated the Pearson correlation coefficient between the reference model and the partial model generated excluding the area being tested.

**Table 1:** Correlation between the reference and partial models.^a^

Area tested for correlation, data excluded from the model generation	Presences	Reference IDW vs. Partial IDW	Reference Maxent vs. Partial Maxent
Area 1	492	−0.2099	0.9888
Area 2	484	−0.0659	0.6564
Area 3	426	−0.325	0.4504
Area 4	523	−0.0558	0.9954

a The reference models were generated using all of the presence data. For each partial model, the presences of the corresponding area were removed, and that window area was then used for the correlation calculation. The correlation between the reference IDW and Maxent models for the entire Iberian Peninsula was 0.1542. Areas as in Figure 1.

## Results

### Species data

Although *Grimmia* mosses grow on the tops of rocks, primarily in open areas, and are relatively conspicuous to nonspecialists, collections of *Grimmia* exist only for 557 of the ~8,100,100 km^2^ cells covering the Iberian Peninsula. For opportunistic sampling localities (Fig. 2A), 86.3% of the collection localities have one or two species, 13.12% have three or four species, and the remaining 0.58% have five or six species. In the systematically sampled localities (Fig. 2B), 44.39% of the localities have one or two species of *Grimmia*, 42.44% have three or four species, and the remaining 13.18% have from five to eight species.

### Richness models

As expected, the reference IDW interpolation model is biased toward those areas that have been systematically collected (Fig. 1B), even though we reduced the power parameter to increase the smoothness of the model. The final surface is highly heterogeneous, a result that may reflect the lack of appropriate surveys for most of the Iberian Peninsula.

**Figure 3:**
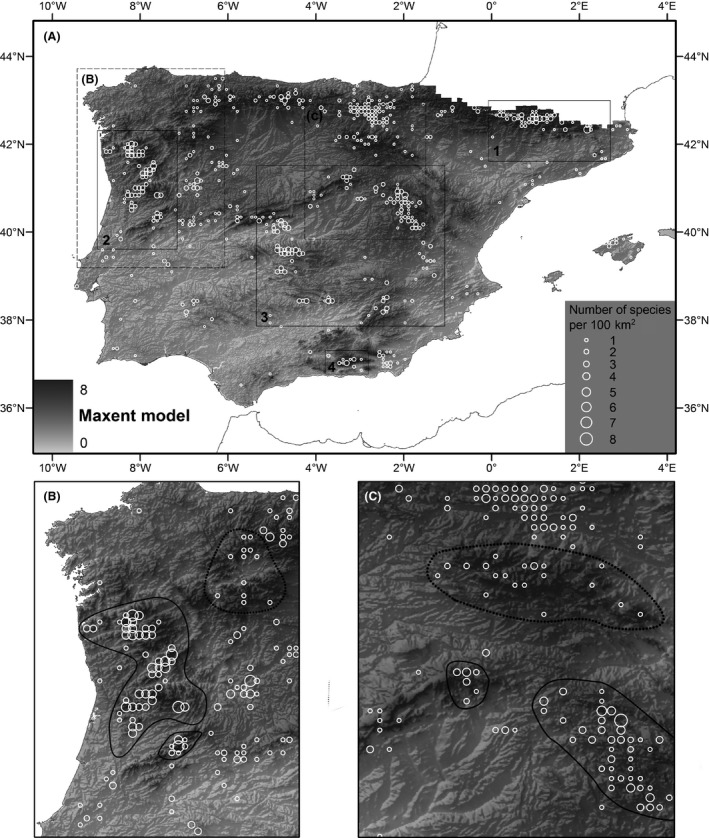
Maxent richness model of *Grimmia* for the Iberian Peninsula. (A) Overlap between the richness model (background) and number of species recorded per 100 km^2^ pixel. (B) and (C) Examples of areas in the Iberian Peninsula for which the richest areas according to the Maxent model match (solid lines) and do not match (dotted lines) the richest areas with more taxa recorded in the natural history collections.

The reference S‐SDM for the genus is shown as the background in Figure 3. The more oceanic areas of the northwestern Iberian Peninsula and the mountain ranges in the interior exhibit conditions that support higher numbers of species. This result coincides with the expected richness pattern based on expert knowledge and the ecology of the resident species (Casas et al. 2006). The average test AUC over the 10 replicates is indicated in Table 2. The AUC values are generally high, except for those species whose main area of distribution is within one of the testing areas. The exclusion of those presences to train the model results in poor AUC values. For example, *G. funalis* is distributed primarily in the Pyrenees (Area 1), and, when this area is eliminated, only seven presences remain to train the model; the sparseness of the remaining training data affects the accuracy of the model (Wisz et al. 2008; Mateo et al. 2010b). A more extreme example would be *G. tergestina*, with 14 of 16 known presences inside Area 3; training the model with the only two presences outside Area 3 explains the low AUC of the model in this area. Finally, the widespread *G. trichophylla* has moderate AUC values, as commonly found for generalist species (McPherson and Jetz 2007; Mateo et al. 2010a). Additionally, preliminary molecular results point that *G. dissimulata* and *G. meridionalis* represent taxa independent from *G. trichophylla*, and the merge of presences of taxa with different environmental requirements would explain the obtained AUC values.

**Table 2:** Maxent test AUC obtained by 10‐fold cross‐validation.^a^

Species	Unique presences	Area 1	Area 2	Area 3	Area 4	Reference model
*G. alpestris*	23	0.9927	0.9934	0.9939	0.9929	0.994
*G. anodon*	8	0.8983	0.9033	0.9033	0.8576	0.9033
*G. caespiticia*	20	0.9818	0.9858	0.9855	0.985	0.9859
*G. crinita*	25	0.9655	0.965	0.9713	0.9703	0.9705
*G. decipiens*	203	0.8585	0.8106	0.8704	0.8592	0.8596
*G. funalis*	19	0.7555	0.9522	0.9539	0.9559	0.9528
*G. hartmanii*	41	0.9426	0.8927	0.9419	0.945	0.9492
*G. laevigata*	130	0.8617	0.7767	0.8751	0.8591	0.8526
*G. lisae*	74	0.8213	0.8396	0.7792	0.8238	0.8165
*G. montana*	178	0.9287	0.8829	0.9364	0.9257	0.9312
*G. orbicularis*	149	0.8695	0.8769	0.8924	0.8611	0.8627
*G. ovalis*	28	0.7952	0.8442	0.8425	0.831	0.8421
*G. pulvinata*	229	0.8176	0.81	0.8552	0.8103	0.806
*G. ramondii*	12	0.963	0.9774	0.9615	0.954	0.9477
*G. reflexidens*	9	0.9778	0.9883	0.9883	0.9621	0.9883
*G. tergestina*	16	0.8135	0.8858	0.5898	0.8589	0.8375
*G. torquata*	9	0.9007	0.9104	0.9617	0.8726	0.9007
*G. trichophylla*	180	0.7447	0.7135	0.752	0.7386	0.7397

a Areas as in Figure 1.

### Comparison of the richness models

The IDW approach produced the map with the lowest values of species richness (mean: 1.8, max: 8 species) while the S‐SDMs approach provided the map with the highest values of species richness (mean: 2.2, max: 10 species).

The correlation between the reference IDW and reference S‐SDMs models was low (*r *=* *0.1542), indicating that the models differ in the information they provide (Table 1). The partial S‐SDMs (i.e., generated without information from a given area) are good predictors of the number of species in that area, as shown by the medium to high correlation values (0.4504 to 0.9954). The lowest correlation is obtained from the tests performed with Area 3 because this area includes 19.16% of the presences. Although the correlation is still high, eliminating this information from the model affects the accuracy of the reference model. In contrast, removing the information from the IDW interpolation has a dramatic effect on the models, as indicated by the low and negative correlation values (−0.0558 to −0.3250).

## Discussion

### Potential richness models

Our results demonstrate for the first time that the combination of individual species models (i.e., S‐SDMs) offers a useful tool for identifying detailed richness patterns for inconspicuous taxa such as bryophytes. The richness patterns generated by S‐SDMs coincide with the expected richness pattern based on expert knowledge and therefore are a powerful tool for basic biodiversity applications (e.g., biogeography and conservation).

Our results confirm that S‐SDMs are less affected by collection biases than are spatial interpolation models and that the information about the richness provided by the S‐SDMs is improved relative to the raw information contained in the NHCs. In this study, we found a correlation between the reference (i.e., created using all of the available presence data) IDW and S‐SDM models of 0.1542, confirming that the richest areas according to the SDMs differ from the areas with more taxa recorded in the NHCs. In the case of the S‐SDMs, we found high correlations between the partial models generated eliminating important pieces of information and the model generated using all of the available information. This result shows that the SDMs investigated were robust and could extract latent information from the data. In contrast, the spatial interpolation models performed poorly and were unable to represent the actual richness (Table 1), although, in some cases, with a good spatial coverage, the implementation of spatial interpolation techniques may increase the predictive accuracy of habitat models, especially for species with loose association with environmental variables (Brotons et al. 2007).

However, SDMs are not free of uncertainty when applied to conservation programs (Rondinini et al. 2006; Carvalho et al. 2010; Underwood et al. 2010; Aranda and Lobo 2011; Mateo et al. 2013a). For example, they may not include all environmental, ecological, and historical factors that affect species distributions (Guisan and Zimmermann 2000). The main caveat of using S‐SDMs to generate species richness patterns is that it tends to overestimate actual species richness (Trotta‐Moreu and Lobo 2010; Dubuis et al. 2011; Mateo et al. 2012). A suggested solution to this problem has been to use macroecological models of species richness to constrain S‐SDMs (see Dubuis et al. 2011; Guisan and Rahbek 2011). Other alternative, “hybrid” models that include basic mechanisms, such as dispersal and demography into SDMs (Boulangeat et al. 2012; Dullinger et al. 2012), have provided more realism and better predictive performance than traditional models (Thuiller et al. 2013). On the other hand, little is known about the performance of S‐SDMs in inconspicuous groups. These results suggest that this distributional proposal should be considered as a preliminary step and that a higher number of database records are needed for the insufficiently surveyed cells.

### Considerations for conservation strategies

Two main types of distribution data are frequently used in conservation planning to obtain distribution maps: observed and predicted data (Rondinini et al. 2006). Additionally, previous studies have discussed the advantages and drawbacks of different approaches that are used to generate species richness patterns as the basis for subsequent conservation measures (Freitag and Jaarsveld 1998; Bombi et al. 2011). Currently, one of the criteria employed in spatial conservation networks is to focus logistic and economic efforts in richness areas (Prendergast et al. 1999; Margules and Pressey 2000; Myers et al. 2000). The most intuitive and simple approach for reserve design is aggregating the NHC data and transferring the resulting information to a geographic space (equivalent to the spatial interpolation model used here). However, the results of this study show that such networks may be strongly biased toward those areas with higher numbers of collections and would not necessarily represent the richest areas. However, it is rarely the case that the areas of interest are sufficiently surveyed, and the existing surveys are certainly not adequate for this purpose in the case of small and inconspicuous organisms and for most geographic areas, including the megadiverse tropics (Cayuela et al. 2009). But, see the limitations described in the first part of this discussion.

Reserve selection is sensitive to the type of distribution data used for the selection (Freitag and Jaarsveld 1998; Wilson et al. 2005; Rondinini et al. 2006). For areas in which detailed surveys have been performed, the richest areas according to the S‐SDM match those with more taxa in the NHCs (Fig. 3B and C, solid lines), whereas such agreement does not occur in the areas for which only opportunistic samples are available (Fig. 3B and C, dashed lines). In general, the richest areas identified by spatial interpolation models coincide with the best‐surveyed areas, indicating that such models represent a restricted approach that excludes poorly sampled areas. In contrast, the S‐SDMs show a richness pattern that is independent of the collection effort and is, therefore, more appropriate for biodiversity patterns studies.

There is a consensus that SDMs are an appropriate tool for management and conservation programs and for the identification of suitable areas for threatened or otherwise rare species (Parviainen et al. 2008, 2009; Williams et al. 2009). Our results show that the S‐SDMs derived from natural history collections improve the incomplete information inherent in the scattered nature of sampling distributions, particularly in the case of opportunistic sampling, by assessing the potential richness of clearly under‐surveyed sites. Therefore, these models contribute additional information that is not obvious from the limited presence data.

We consider that S‐SDMs should be used to produce potential maps of species richness when information is limited and different types of distribution data are available (opportunistically sampled vs. systematically sampled localities). Consequently, S‐SDMs could be highly useful for reserve design at the national scale. This conclusion is in agreement with several studies that stressed that predictive models can effectively address the problem of insufficient field survey and museum data (Loiselle et al. 2003; Maes et al. 2005; Rodríguez et al. 2007; Sérgio et al. 2007b; Braunisch and Suchant 2010; Costa et al. 2010; Mateo et al. 2013a) and offer benefits for conservation prioritization (Elith and Leathwick 2009). Using models to predict distributions is also likely to become increasingly important as climate change and other dynamic processes are incorporated into conservation planning efforts (Rondinini et al. 2006; Underwood et al. 2010). Although the S‐SDMS seems to outperform the IDW approach, both approaches combined may provide improved information for directing future efforts to conserve these inconspicuous taxa and targeting areas for monitoring and management. Sánchez‐Fernández et al. (2011) demonstrated that species distribution models, combined with a survey effort map, might be used to select the location of future surveys by prioritizing those species‐rich areas with a low level of sampling effort. Here, the IDW output could be seen as a proposal of a survey effort map which overlaid with the S‐SDM output could allow us to locate those areas where more sampling effort is necessary. Another possibility is the combination of S‐SDMs with macroecological models (Guisan and Rahbek 2011).

## Conclusions

From our study, we can conclude that (1) using a modeling approach based on the combination of individual species models (stacking species distribution models) allowed the identification of detailed richness patterns for inconspicuous taxa such as bryophytes. (2) Stacking species distribution models are less affected by collection biases or types of distribution data than are spatial interpolation models (traditional approach) and therefore could be highly useful for conservation purposes. (3) Spatial interpolation models may provide a complementary view to the modeling approach.

For the Iberian Peninsula, further studies should aim at refining this modeling approach with more taxonomic groups within bryophytes, and also with additional geographic information that would inform about new areas for conservation for inconspicuous groups such as bryophytes.

## Conflict of Interest

None declared.

## Supporting information


**Data S1.** Simplified grid specifying the acidic/basic nature of each pixel used.

## References

[ece31796-bib-0001] Algar, A. C. , H. M. Kharouba , E. R. Young , and J. T. Kerr . 2009 Predicting the future of species diversity: macroecological theory, climate change, and direct tests of alternative forecasting methods. Ecography 32:22–33.

[ece31796-bib-0002] Anderson, S. 2002 Identifying important plant areas. Plantlife International, London.

[ece31796-bib-0003] Anderson, R. P. , and I. Jr Gonzalez . 2011 Species‐specific tuning increases robustness to sampling bias in models of species distributions: An implementation with Maxent. Ecol. Model. 222:2796–2811.

[ece31796-bib-0004] Aranda, S. C. , and J. M. Lobo . 2011 How well does presence‐only‐based species distribution modelling predict assemblage diversity? A case study of the Tenerife flora. Ecography 34:31–38.

[ece31796-bib-0005] Austin, G. E. , C. J. Thomas , D. C. Houston , and D. B. A. Thompson . 1996 Predicting the spatial distribution of Buzzard Buteo buteo nesting areas using a geographical information system and remote sensing. J. Appl. Ecol. 33:1541–1550.

[ece31796-bib-0006] Boitani, L. , L. Maiorano , D. Baisero , A. Falcucci , P. Visconti , and C. Rondinini . 2011 What spatial data do we need to develop global mammal conservation strategies? Philos. Trans. R. Soc. Lond. B Biol. Sci. 366:2623–2632. 21844041 10.1098/rstb.2011.0117 PMC3140738

[ece31796-bib-0007] Bombi, P. , L. Luiselli , and M. D'Amen . 2011 When the method for mapping species matters: defining priority areas for conservation of African freshwater turtles. Divers. Distrib. 17:581–592.

[ece31796-bib-0008] Boulangeat, I. , D. Gravel , and W. Thuiller . 2012 Accounting for dispersal and biotic interactions to disentangle the drivers of species distributions and their abundances. Ecol. Lett. 15:584–593. 22462813 10.1111/j.1461-0248.2012.01772.x PMC3999639

[ece31796-bib-0009] Braunisch, V. , and R. Suchant . 2010 Predicting species distributions based on incomplete survey data: the trade‐off between precision and scale. Ecography 33:826–840.

[ece31796-bib-0010] Brooks, T. M. , R. A. Mittermeier , C. G. Mittermeier , G. A. B. Fonseca , A. B. Rylands , W. R. Konstant , et al. 2002 Habitat loss and extinction in the hotspots of biodiversity. Conserv. Biol. 16:909–923.

[ece31796-bib-0011] Brotons, L. , S. Herrando , and M. Pla . 2007 Updating bird species distribution at large spatial scales: applications of habitat modelling to data from long‐term monitoring programs. Divers. Distrib. 13:276–288.

[ece31796-bib-0012] Butchart, S. H. M. , M. Walpole , B. Collen , A. van Strien , J. P. W. Scharlemann , R. E. A. Almond , et al. 2010 Global biodiversity: indicators of recent declines. Science 328:1164–1168. 20430971 10.1126/science.1187512

[ece31796-bib-0013] Calabrese, J. M. , G. Certain , C. Kraan , and C. F. Dormann . 2014 Stacking species distribution models and adjusting bias by linking them to macroecological models. Glob. Ecol. Biogeogr. 23:99–112.

[ece31796-bib-0014] Carvalho, M. R. , F. A. Bockmann , D. S. Amorim , C. R. F. Brandão , M. Vivo , J. L. Figueiredo , et al. 2007 Taxonomic impediment or impediment to taxonomy? A commentary on systematics and the cybertaxonomic‐automation paradigm. Evol. Biol. 34:140–143.

[ece31796-bib-0015] Carvalho, S. B. , J. C. Brito , R. L. Pressey , E. Crespo , and H. P. Possingham . 2010 Simulating the effects of using different types of species distribution data in reserve selection. Biol. Conserv. 143:426–438.

[ece31796-bib-0016] Casas, C. , M. Brugués , R. M. Cros , and C. Sérgio . 2006 Handbook of mosses of the Iberian Peninsula and the Balearic Islands. Institut d'Estudis Catalans. Secció de Ciències Biològiques, Barcelona.

[ece31796-bib-0017] Cayuela, L. , D. J. Golicher , A. C. Newton , M. Kolb , F. S. Alburquerque , E. J. M. M. Arets , et al. 2009 Species distribution modeling in the tropics: problems, potentialities, and the role of biological data for effective species conservation. Trop. Conserv. Sci. 2:319–352.

[ece31796-bib-0018] Costa, G. C. , C. Nogueira , R. B. Machado , and G. R. Colli . 2010 Sampling bias and the use of ecological niche modeling in conservation planning: a field evaluation in a biodiversity hotspot. Biodivers. Conserv. 19:883–899.

[ece31796-bib-0019] Désamoré, A. , B. Laenen , M. Stech , B. Papp , L. Hedenäs , R. G. Mateo , et al. 2012 How do temperate bryophytes face the challenge of a changing environment? Lessons from the past and predictions for the future. Glob. Change Biol. 18:2915–2924. 10.1111/j.1365-2486.2012.02752.x 24501067

[ece31796-bib-0020] Dubuis, A. , J. Pottier , V. Rion , L. Pellissier , J.‐P. Theurillat , and A. Guisan . 2011 Predicting spatial patterns of plant species richness: a comparison of direct macroecological and species stacking modelling approaches. Divers. Distrib. 17:1122–1131.

[ece31796-bib-0021] Dullinger, S. , A. Gattringer , W. Thuiller , D. Moser , N. E. Zimmermann , A. Guisan , et al. 2012 Extinction debt of high‐mountain plants under twenty‐first‐century climate change. Nat. Clim. Chang. 2:619–622.

[ece31796-bib-0022] Elith, J. , and J. Leathwick . 2009 The contribution of species distribution modelling to conservation prioritization Pp. 70–93 *in* Moilanen A., Wilson K. A., Possingham H. P., eds. Spatial conservation prioritization. Quantitative methods and computational tools. Oxford Univ. Press, Oxford.

[ece31796-bib-0023] Elith, J. , C. H. Graham , R. P. Anderson , M. Dudík , S. Ferrier , A. Guisan , et al. 2006 Novel methods improve prediction of species' distributions from occurrence data. Ecography 29:129–151.

[ece31796-bib-0024] Elith, J. , S. J. Phillips , T. Hastie , M. Dudík , Y. E. Chee , and C. J. Yates . 2011 A statistical explanation of MaxEnt for ecologists. Divers. Distrib. 17:43–57.

[ece31796-bib-0025] Ferrier, S. 2002 Mapping spatial pattern in biodiversity for regional conservation planning: where to from here? Syst. Biol. 51:331–363. 12028736 10.1080/10635150252899806

[ece31796-bib-0026] Ferrier, S. , and A. Guisan . 2006 Spatial modelling of biodiversity at the community level. J. Appl. Ecol. 43:393–404.

[ece31796-bib-0027] Franklin, J. , and J. A. Miller . 2009 Mapping species distributions: spatial inference and prediction. Cambridge Univ. Press, Cambridge, U.K..

[ece31796-bib-0028] Freitag, S. , and A. S. V. Jaarsveld . 1998 Sensitivity of selection procedures for priority conservation areas to survey extent, survey intensity and taxonomic knowledge. Proc. R. Soc. B Biol. Sci. 265:1475–1482.

[ece31796-bib-0029] Garcillán, P. P. , and E. Ezcurra . 2011 Sampling procedures and species estimation: testing the effectiveness of herbarium data against vegetation sampling in an oceanic island. J. Veg. Sci. 22:273–280.

[ece31796-bib-0030] Gaubert, P. , M. Papes , and A. T. Peterson . 2006 Natural history collections and the conservation of poorly known taxa: Ecological niche modeling in central African rainforest genets (Genetta spp.). Biol. Conserv. 130:106–117.

[ece31796-bib-0031] Geffert, J. L. , J.‐P. Frahm , W. Barthlott , and J. Mutke . 2013 Global moss diversity: spatial and taxonomic patterns of species richness. J. Bryol. 35:1–11.

[ece31796-bib-0032] Gelfand, A. E. , A. M. Schmidt , S. Wu , J. A. Jr Silander , A. Latimer , and A. G. Rebelo . 2005 Modelling species diversity through species level hierarchical modelling. Appl. Stat. 54:1–20.

[ece31796-bib-0033] Graham, C. H. , and R. J. Hijmans . 2006 A comparison of methods for mapping species ranges and species richness. Glob. Ecol. Biogeogr. 15:578–587.

[ece31796-bib-0034] Graham, C. H. , S. Ferrier , F. Huettman , C. Moritz , and A. T. Peterson . 2004 New developments in museum‐based informatics and applications in biodiversity analysis. Trends Ecol. Evol. 19:497–503. 16701313 10.1016/j.tree.2004.07.006

[ece31796-bib-0035] Guisan, A. , and C. Rahbek . 2011 SESAM – a new framework integrating macroecological and species distribution models for predicting spatio‐temporal patterns of species assemblages. J. Biogeogr. 38:1433–1444.

[ece31796-bib-0036] Guisan, A. , and N. E. Zimmermann . 2000 Predictive habitat distribution models in ecology. Ecol. Model. 135:147–186.

[ece31796-bib-0037] Hafernik, J. Jr . 1992 Threats to invertebrate biodiversity: implications for conservation strategies *in* Fiedler P. and Jain S., eds. Conservation biology. Chapman and Hall, London.

[ece31796-bib-0038] Hedenäs, L. 2007 Global diversity patterns among pleurocarpous mosses. Bryologist 110:319–331.

[ece31796-bib-0039] Hernandez, P. A. , C. H. Graham , L. L. Master , and D. L. Albert . 2006 The effect of sample size and species characteristics on performance of different species distribution modeling methods. Ecography 29:773–785.

[ece31796-bib-0040] Hernandez‐Stefanoni, J. L. , and R. Ponce‐Hernandez . 2006 Mapping the spatial variability of plant diversity in a tropical forest: comparison of spatial interpolation methods. Environ. Monit. Assess. 117:307–334. 16917715 10.1007/s10661-006-0885-z

[ece31796-bib-0041] Hijmans, R. J. , S. E. Cameron , J. L. Parra , P. G. Jones , and A. Jarvis . 2005 Very high resolution interpolated climate surfaces for global land areas. Int. J. Climatol. 25:1965–1978.

[ece31796-bib-0042] Hunter, M. L. , and S. L. Webb . 2002 Enlisting taxonomists to survey poorly known Taxa for biodiversity conservation: a Lichen case study. Conserv. Biol. 16:660–665.

[ece31796-bib-0043] Infante, M. , and P. Heras . 2012 Red preliminar de Áreas Importantes para los Briófitos (IBrA) Pp. 215–287 *in* Garilleti R., Albertos B. (Coords.). Atlas y Libro Rojo de los Briófitos Amenazados de España. Ed. Organismo Autónomo Parques Nacionales, Madrid.

[ece31796-bib-0044] Jeschke, J. M. , and D. L. Strayer . 2008 Usefulness of bioclimatic models for studying climate change and invasive species. Ann. N. Y. Acad. Sci. 1134:1–24. 18566088 10.1196/annals.1439.002

[ece31796-bib-0045] Kruijer, J. D. , N. Raes , and M. Stech . 2010 Modelling the distribution of the moss species *Hypopterygium tamarisci* (Hypopterygiaceae, Bryophyta) in Central and South America. Nova Hedwigia 91:399–420.

[ece31796-bib-0046] Lobo, J. M. , A. Jiménez‐Valverde , and R. Real . 2008 AUC: a misleading measure of the performance of predictive distribution models. Glob. Ecol. Biogeogr. 17:145–151.

[ece31796-bib-0047] Loiselle, B. , C. A. Howell , C. H. Graham , J. M. Goerck , T. Brooks , K. G. Smith , et al. 2003 Avoiding pitfalls of using species distributions models in conservation planning. Conserv. Biol. 17:1591–1600.

[ece31796-bib-0048] Maes, D. , D. Bauwens , L. D. Bruyn , A. Anselin , G. Vermeersch , W. V. Landuyt , et al. 2005 Species richness coincidence: conservation strategies based on predictive modelling. Biodivers. Conserv. 14:1345–1364.

[ece31796-bib-0049] Margules, C. R. , and R. L. Pressey . 2000 Systematic conservation planning. Nature 405:243–253. 10821285 10.1038/35012251

[ece31796-bib-0050] Mateo, R. G. , Á. M. Felicísimo , and J. Muñoz . 2010a Effects of the number of presences on reliability and stability of MARS species distribution models: the importance of regional niche variation and ecological heterogeneity. J. Veg. Sci. 21:908–922.

[ece31796-bib-0051] Mateo, R. G. , B. C. Thomas , Á. M. Felicísimo , and J. Muñoz . 2010b Profile or group discriminative techniques? Generating reliable species distribution models using pseudo‐absences and target‐group absences from natural history collections. Divers. Distrib. 16:84–94.

[ece31796-bib-0052] Mateo, R. G. , Á. M. Felicísimo , and J. Muñoz . 2011 Modelos de distribución de especies: Una revisión sintética. Species distributions models: A synthetic revision. Rev. Chil. Hist. Nat. 84:217–240.

[ece31796-bib-0053] Mateo, R. G. , A. M. Felicísimo , J. Pottier , A. Guisan , and J. Muñoz . 2012 Do stacked species distribution models reflect altitudinal diversity patterns? PLoS ONE 7:1–9. 10.1371/journal.pone.0032586 PMC3292561 22396782

[ece31796-bib-0054] Mateo, R. G. , M. Estrella , Á. M. Felicísimo , J. Muñoz , and A. Guisan . 2013a A new spin on a compositionalist predictive modelling framework for conservation planning: A tropical case study in Ecuador. Biol. Conserv. 160:150–161.

[ece31796-bib-0055] Mateo, R. G. , A. Vanderpoorten , J. Muñoz , B. Laenen , and A. Désamoré . 2013b Modeling species distributions from heterogeneous data for the biogeographic regionalization of the European bryophyte flora. PLoS ONE 8:1–11. 10.1371/journal.pone.0055648 PMC3569459 23409015

[ece31796-bib-0056] McPherson, J. M. , and W. Jetz . 2007 Effects of species' ecology on the accuracy of distribution models. Ecography 30:135–151.

[ece31796-bib-0057] Muñoz, J. , and F. Pando , (2000).A world synopsis of the genus Grimmia (Musci, Grimmiaceae). Monographs in systematic botany from the Missouri botanical garden (Vol. 83). Missouri Botanical Garden Press, St. Louis, MO.

[ece31796-bib-0058] Muñoz, J. , Á. M. Felicísimo , F. Cabezas , A. R. Burgaz , and I. Martínez . 2004 Wind as a long‐distance dispersal vehicle in the Southern Hemisphere. Science 304:1144–1147. 15155945 10.1126/science.1095210

[ece31796-bib-0059] Muñoz, J. , H. Hespanhol , K. Cezón , and A. Séneca . 2009 Grimmia horrida (Grimmiaceae) a new species from the Iberian Peninsula. Bryologist 112:325–328.

[ece31796-bib-0060] Myers, N. , R. A. Mittermeier , C. G. Mittermeier , G. A. B. Fonseca , and J. Kent . 2000 Biodiversity hotspots for conservation priorities. Nature 403:853–858. 10706275 10.1038/35002501

[ece31796-bib-0061] Newbold, T. 2010 Applications and limitations of museum data for conservation and ecology, with particular attention to species distribution models. Prog. Phys. Geogr. 34:3–22.

[ece31796-bib-0062] Oliver, I. , and A. J. Beattie . 1993 A possible method for the rapid assessment of biodiversity. Conserv. Biol. 7:562–568.

[ece31796-bib-0063] Papp, B. 2008 Selection of important bryophyte areas in Hungary. Folia Cryptogam. Est. 44:101–111.

[ece31796-bib-0064] Parviainen, M. , M. Luoto , T. Ryttäri , and R. K. Heikkinen . 2008 Modelling the occurrence of threatened plant species in taiga landscapes: Methodological and ecological perspectives. J. Biogeogr. 35:1888–1905.

[ece31796-bib-0065] Parviainen, M. , M. Marmion , M. Luoto , W. Thuiller , and R. K. Heikkinen . 2009 Using summed individual species models and state‐of‐the‐art modelling techniques to identify threatened plant species hotspots. Biol. Conserv. 142:2501–2509.

[ece31796-bib-0066] Peterson, A. T. , M. Papeş , and J. Soberón . 2008 Rethinking receiver operating characteristic analysis applications in ecological niche modeling. Ecol. Model. 213:63–72.

[ece31796-bib-0067] Phillips, S. J. , and M. Dudík . 2008 Modeling of species distributions with Maxent: new extensions and a comprehensive evaluation. Ecography 31:161–175.

[ece31796-bib-0068] Phillips, S. J. , R. P. Anderson , and R. E. Schapired . 2006 Maximum entropy modeling of species geographic distributions. Ecol. Model. 190:231–259.

[ece31796-bib-0069] Prendergast, J. R. , R. M. Quinn , and J. H. Lawton . 1999 The gaps between theory and practice in selecting nature reserves. Conserv. Biol. 13:484–492.

[ece31796-bib-0070] Ricklefs, R. 2004 A comprehensive framework for global patterns in biodiversity. Ecol. Lett. 7:1–15.

[ece31796-bib-0071] Rodríguez, J. P. , L. Brotons , J. Bustamante , and J. Seoane . 2007 The application of predictive modelling of species distribution to biodiversity conservation. Divers. Distrib. 13:243–251.

[ece31796-bib-0072] Rondinini, C. , K. A. Wilson , L. Boitani , H. Grantham , and H. P. Possingham . 2006 Tradeoffs of different types of species occurrence data for use in systematic conservation planning. Ecol. Lett. 9:1136–1145. 16972877 10.1111/j.1461-0248.2006.00970.x

[ece31796-bib-0073] Roux, P. C. , R. Virtanen , R. K. Heikkinen , and M. Luoto . 2012 Biotic interactions affect the elevational ranges of high‐latitude plant species. Ecography 35:1048–1056.

[ece31796-bib-0074] Sánchez‐Fernández, D. , J. M. Lobo , P. Abellán , and A. Millán . 2011 How to identify future sampling areas when information is biased and scarce: An example using predictive models for species richness of Iberian water beetles. J. Nat. Conserv. 19:54–59.

[ece31796-bib-0075] Schumacker, R. , and P. Martiny . 1995 Threatened bryophytes in Europe including Macaronesia Pp. 29–193 *in* ECCB , ed. Red data book of European bryophytes. ECCB, Trondheim.

[ece31796-bib-0076] Sérgio, C. , and D. Draper . 2002 How to evaluate species when distribution is poorly understood. The use of predictive studies for Iberian Bryophytes. Port. Acta Biol. 20:37–47.

[ece31796-bib-0077] Sérgio, C. , M. Brugués , R. M. Cros , C. Casas , and C. Garcia . 2007a The 2006 Red List and an updated checklist of bryophytes of the Iberian Peninsula (Portugal, Spain, and Andorra). Lindbergia 31:109–125.

[ece31796-bib-0078] Sérgio, C. , R. Figueira , D. Draper , R. Menezes , and A. J. Sousa . 2007b Modelling bryophyte distribution based on ecological information for extent of occurrence assessment. Biol. Conserv. 135:341–351.

[ece31796-bib-0079] Sérgio, C. , R. Figueira , and R. Menezes . 2011 Modeling the distribution of Sematophyllum substrumulosum (Hampe) E. Britton as a signal of climatic changes in Europe Pp. 427–440 *in* Tuba Z., Slack N. G., Stark L. R., eds. Bryophyte ecology and climate change. Cambridge Univ. Press, Cambridge.

[ece31796-bib-0080] Sérgio, C. , C. A. Garcia , H. Hespanhol , C. Vieira , S. Stow , and D. Long . 2012 Bryophyte diversity in the Peneda‐Gerês National Park (Portugal): selecting Important Plant Areas (IPA) based on a new survey and past records. Bot. Complut. 36:39–50.

[ece31796-bib-0081] Shaw, A. J. , C. J. Cox , and B. Goffinet . 2005 Global patterns of moss diversity: taxonomic and molecular inference. Taxon 54:337–352.

[ece31796-bib-0082] Thomas, C. D. , A. Cameron , R. E. Green , M. Bakkenes , L. J. Beaumont , Y. C. Collingham , et al. 2004 Extinction risk from climate change. Nature 427:145–148. 14712274 10.1038/nature02121

[ece31796-bib-0083] Thuiller, W. , T. Münkemüller , S. Lavergne , D. Mouillot , N. Mouquet , K. Schiffers , et al. 2013 A road map for integrating eco‐evolutionary processes into biodiversity models. Ecol. Lett. 16:94–105. 23679011 10.1111/ele.12104 PMC3790307

[ece31796-bib-0084] Trotta‐Moreu, N. , and J. M. Lobo . 2010 Deriving the species richness distribution of geotrupinae (Coleoptera: Scarabaeoidea) in Mexico from the overlap of individual model predictions. Environ. Entomol. 39:42–49. 20146838 10.1603/EN08179

[ece31796-bib-0085] Underwood, J. G. , C. D'Agrosa , and L. R. Gerber . 2010 Identifying conservation areas on the basis of alternative distribution data sets. Conserv. Biol. 24:162–170. 19659686 10.1111/j.1523-1739.2009.01303.x

[ece31796-bib-0086] Vanderpoorten, A. , and B. Goffinet . 2009 Introduction to bryophytes. Cambridge Univ. Press, London.

[ece31796-bib-0087] Vanderpoorten, A. , A. Sotiaux , and P. Engels . 2005 A GIS‐based survey for the conservation of bryophytes at the landscape scale. Biol. Conserv. 121:189–194.

[ece31796-bib-0088] Warren, D. L. , and S. N. Seifert . 2011 Ecological niche modeling in Maxent: the importance of model complexity and the performance of model selection criteria. Ecol. Appl. 21:335–342. 21563566 10.1890/10-1171.1

[ece31796-bib-0089] Williams, J. N. , C. Seo , J. Thorne , J. K. Nelson , S. Erwin , J. M. O'Brien , et al. 2009 Using species distribution models to predict new occurrences for rare plants. Divers. Distrib. 15:565–576.

[ece31796-bib-0090] Wilson, K. A. , M. I. Westphal , H. P. Possingham , and J. Elith . 2005 Sensitivity of conservation planning to different approaches to using predicted species distribution data. Biol. Conserv. 122:99–112.

[ece31796-bib-0091] Wisz, M. S. , R. J. Hijmans , J. Li , A. T. Peterson , C. H. Graham , and A. Guisan . 2008 Effects of sample size on the performance of species distribution models. Divers. Distrib. 14:763–773.

