# Supplementary material for: How to describe species richness patterns for bryophyte conservation?
Source: Ecol Evol. 2015 Oct 28;5(23):5443–55. doi: 10.1002/ece3.1796 (PMC4813098; doi:10.1002/ece3.1796)
Supplement: Supplementary file 1 — Data S1. Simplified grid specifying the acidic/basic nature of each pixel used. [file ECE3-5-5443-s001.pdf]

This file is an ASCII-formatted text file in ESRI ASCII raster format and has the information of soil acidity/alkalinity (categorical variable: 0 – acid; 1-basic) for the Iberian Peninsula that was derived from the European Soil Database v2.0 (http://eusoils.jrc.ec.europa.eu/ESDB_Archive/ESDB_data_1k_raster_intro/ESDB_1k_raster_data_intro.html).
